# The Impact of Lockdown, Patient Classification, and the Large-Scale Case Screening on the Spread of the Coronavirus Disease 2019 (COVID-19) in Hubei

**DOI:** 10.1155/2022/8920117

**Published:** 2022-05-06

**Authors:** Shengtao Wang, Yan Li, Ximei Wang, Yuanyuan Zhang, Yiyi Yuan, Yong Li

**Affiliations:** ^1^School of Information and Mathematics, Yangtze University, Jingzhou 434023, China; ^2^School of Foreign Studies, Yangtze University, Jingzhou 434023, China; ^3^Viterbi School of Engineering, University of Southern California, Los Angeles CA 90007, USA

## Abstract

The coronavirus disease (COVID-19) which emerged in Wuhan, China, in December 2019, is widely controlled now in China. However, the global epidemic is still severe. To study and comment on Hubei's approaches for responding to the disease, the paper considered some factors such as suspected cases (part of them are influenza patients or common pneumonia patients, etc.), quarantine, patient classification (three types), clinically diagnosed cases, and lockdown of Wuhan and Hubei. After that, the paper established an *SELIHR* model based on the surveillance data of Hubei published by the Hubei Health Commission from 10 January 2020 to 30 April 2020 and used the fminsearch optimization method to estimate the optimal parameters of the model. We obtained the basic reproduction number *ℛ*_0_ = 3.1571 from 10 to 22 January. *ℛ*_0_ was calculated as 2.0471 from 23 to 27 January. From 28 January to 30 April, *ℛ*_0_ = 1.5014. Through analysis, it is not hard to find that the patients without classification during the period of confirmed cases will result in the cumulative number of cases in Hubei to increase. In addition, regarding the lockdown measures implemented by Hubei during the epidemic, our simulations also show that if the lockdown time of either Hubei or Wuhan is advanced, it will effectively curb the spread of the epidemic. If the lockdown measures are not taken, the total cumulative number of cases will increase substantially. From the results of the study, it can be concluded that the lockdown, patient classification, and the large-scale case screening are essential to slow the spread of COVID-19, which can provide references for other countries or regions.

## 1. Introduction

In December 2019, a novel coronavirus emerged in Wuhan, Hubei province. Then the World Health Organization (WHO) formally named the novel coronavirus COVID-19 on 11 February 2020 [1]. Coincided with transportation during the Spring Festival (called Chunyun in China), China took strict prevention measures to control the spread of the outbreak like closing all premises, imposing certain restrictions on the passage of the provinces, and paying attention to personal hygiene and other actions. On 23 January 2020, the authorities in Wuhan announced the lockdown of the city and closed all expressways leaving Wuhan [2]. After that, 12 cities and an autonomous prefecture took the same measures, and all people in Hubei province were advised to quarantine at home. When the situation got better, the traffic control in Hubei province was orderly cancelled except for Wuhan on 25 March. People can only rely on the Hubei health green code to other places smoothly [2]. After 76 days of continuous lockdown, the government in Wuhan gradually lifted its lockdown and people returned to their normal life. On 16 April 2020, the number of new cases in Hubei province decreased to 0 [2, 3]. On 26 April 2020, all cases of COVID-19 hospitalization in Wuhan city were cleared [2, 4]. On 11 May 2020, Wuhan conducted a 10-day city-wide nucleic acid detection of all its residents to determine the current epidemic situation [5].

COVID-19 spreads mainly through respiratory droplets, contact, aerosols, feces-mouth, and other forms [6]. The population can be easily infected in direct or indirect ways when they are exposed to long-term closed environments. The source of infection found so far is mainly in patients infected with COVID-19 and patients with asymptomatic infections. Based on current epidemiological investigations and findings, the incubation lasts 1 to 14 days theoretically, but usually 3 to 7 days in reality [6].

Patients who were infected with COVID-19 had main symptoms like fever, dry cough, and fatigue, and a small number of patients were accomplished with nasal congestion, runny nose, sore throat, myalgia, diarrhea, and other symptoms [6]. During the treatment of confirmed cases, the cases were divided into three types [7]: mild, severe, and critical. For the three cases, there were different clinical classification standards. Patients who had light clinical symptoms were mild, and their images did not show signs of pneumonia. Severe patients appeared with obvious respiratory distress with drowsiness, food refusal, and other symptoms. Critical cases referred to patients who appeared with respiratory failure, shock, and other serious life-threatening characteristics. However, it is still unknown whether such classification measures can have an impact on the suppression of the epidemic. Therefore, what impact it will bring is one of the main purposes of this research.

It is well known that the basic reproduction number can measure the level of the epidemic, and the magnitude of *ℛ*_0_ allows one to determine the amount of effort which is necessary either to prevent an epidemic or to eliminate infection from a population [8], so it is crucial to estimate *ℛ*_0_ for a given disease in a particular population. Until now, many researchers have paid attention to this outbreak and have some results [9–46] about the basic reproduction numbers in Wuhan, Hubei, or China which are summarized in Table 1.

Since the outbreak of the epidemic, scholars at home and abroad have done a lot of work and breakthroughs. Mizumoto et al. [11], Jung et al. [12], Riou et al. [13], Li et al. [14], and Zhao et al. [15] made a certain assessment of the transmission potential and prevalence of COVID-19 at the beginning of the outbreak and believed that COVID-19 had a risk of global spread. Anderson et al. [47] argued that contact tracing can help contain the spread of COVID-19 but is still needed to control imported cases. Some papers [9, 16–25] have studied travel bans or isolation measures such as lockdown, among which Collins and Duffy [9] and Pang et al. [17] have discussed the effects of multiple control measures, and their studies suggested that in a series of measures, isolation plays the most important role. Zhang et al. [21], Sun et al. [16], and Wang et al. [26] also considered the impact of medical resource capacity on the spread of the epidemic. In addition, similar to the patient classification in Hubei, the Indonesian government also classified COVID-19 patients into three types, people under monitoring (ODP), patients under surveillance (PDP), and confirmed patients. Artiono et al. [48] included the three types in the established model to study. However, there are still few papers studying patient classification combined with mathematical models.

As of 16 April, the cumulative reported cases of COVID-19 in Hubei province were 68,128, including 50,333 cases in Wuhan, and there were 4,512 people dying of the disease (3,869 in Wuhan) [49]. The number of patients in Hubei province was cleared at the end of April 2020 [4]. In the following nearly two years, there has never been a large-scale outbreak in Hubei, and only a few sporadic cases have occurred until now. Compared with the global epidemic, undoubtedly, the prevention and control measures taken by Hubei have achieved great success. Considering the actual epidemic situation and various response measures taken by the government in Hubei, a dynamic model has been established in this paper to study the inhibitory effect of some kinds of control factors: whether to lock down the city, timely lockdown, patient classification, and large-scale case screening. Through carefully studying the successful case of epidemic prevention in Hubei, the purpose of this paper is to provide some references for the prevention and control of the COVID-19 epidemic in other countries or regions with similar characteristics.

This paper is divided into five sections: Introduction opens with the origin, epidemiology, diagnosis of COVID-19, and the situation in Hubei province; Method develops the study by introducing the method of the study, with which we build a model of COVID-19 based on the real situation and construct the differential equations. We collect the surveillance data and choose the fminsearch optimization method to estimate parameters. The results of the model-based estimates, basic reproduction numbers, and sensitivity analysis are provided in Results. And finally, in Discussion and Conclusion, we discuss the results and give the conclusion of the study.

## 2. Method

### 2.1. The COVID-19 Model

With the outbreak of COVID-19 in China, the decisive measures the Chinese government has taken effectively restrict the movement of people across provinces, especially in Hubei province [23]. On this account, we propose a compartmental dynamics model, and we divide COVID-19-related populations into nine epidemiological subgroups: susceptible (*S*); exposed (*E*); suspected and undetected (*L*); removed, such as influenza patients or common pneumonia patients (*C*); clinically diagnosed cases (*I*); mild patients (*H*_1_); severe patients (*H*_2_); critical patients (*H*_3_); and recovered (*R*). The total population size is denoted by *N* = *S* + *E* + *L* + *I* + *H* + *R* (*H* here is the total number of confirmed cases, *H* = *H*_1_ + *H*_2_ + *H*_3_). In order to make the model more suitable for the local situation in Hubei province, we put forward the following conditions:

1. Since we have only studied for a relatively short period of time, we ignore natural births and deaths, but we do not ignore deaths from COVID-19
2. Asymptomatic transmission is a form of COVID-19 disease transmission [7]. For example, a person who contracts COVID-19 from someone with few or without symptoms may still be infected [50]. If we put the above examples into the model: exposed (*E*), suspected and undetected (*L*), clinically diagnosed cases (*I*), and confirmed cases (*H*), they might lead susceptible patients (*S*) to infect COVID-19 with different infection probabilities
3. Novel coronavirus nucleic acid test-negative patients and a small number of COVID-19 self-healing patients from suspected and undetected (*L*) will still have a certain possibility of contacting COVID-19, so some people in this group will become susceptible (*S*)
4. The city of Wuhan in Hubei province was locked down on 23 January 2020, and other areas in Hubei province were also locked down one after another. Xiangyang city, the last city in Hubei province, was locked down on 27 January 2020. Considering that the symptoms of COVID-19 are very similar to those of influenza patients or common pneumonia patients, etc. In addition, some suspected and undetected (*L*) are actually just influenza patients or common pneumonia patients, etc. So, we introduce (*β*_*i*_, *i* = 1, 2) the following. The *β*_1_ represents the transmission rate of COVID-19, and *β*_2_ represents the transmission rate of influenza or common pneumonia, etc.

(1) $$\begin{array}{c} \beta_{i}=\left\{\begin{array}{c} \beta_{ia},10-22\text{ January }2020,\text{Wuhan was not locked down},\\ \beta_{ib},23-27\text{ January }2020,\text{the cities in Hubei have been locked down},\\ \beta_{ic},28\text{ January}‐30\text{ April }2020,\text{after Hubei was locked down} \end{array}\right. \end{array}$$

(5) The probability of medical personnel contracting COVID-19 (*k*, *q*) before and after the lockdown in Hubei is different. The risk before the lockdown should be higher than that after it was taken, which is due to the initial shortage of medical supplies and the lack of understanding of the epidemic propagation mode. The lockdown in Hubei and the outbreak of the initial disease had led to a serious shortage of viral nucleic acid test kits, which also affected the time required for diagnosis (1/*σ*) to a certain extent. Here, we put *τ* = (*k*, *q*, *σ*) into a parameter set:

(2) $$\begin{array}{c} \tau =\left\{\begin{array}{c} \tau_{a},10-22\text{ January }2020,\text{Wuhan was not locked down},\\ \tau_{b},23-27\text{ January }2020,\text{the cities in Hubei have been locked down},\\ \tau_{c},28\text{ January}‐30\text{ April }2020,\text{after Hubei was locked down} \end{array}\right. \end{array}$$

(6) From the beginning of the lockdown in Wuhan to the lockdown in Xiangyang, some people in Hubei were out of the province, and some people were not susceptible compared with others in this epidemic. We remove these people from the susceptible (*S*) compartment:

(3) $$\begin{array}{c} \varphi =\left\{\begin{array}{c} \varphi_{a\;,}\text{ some people who left Wuhan city on }23\text{ January }2020,\\ \varphi_{b\;,}\text{ some people who left Hubei province on }27\text{ January }2020 \end{array}\right. \end{array}$$

**Figure 1:**
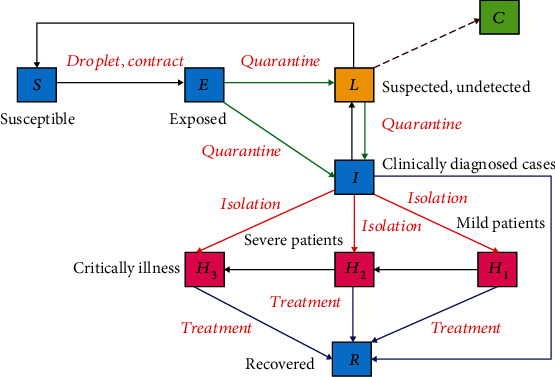
Flow chart of compartments of the COVID-19 model.

Based on the above analysis, a schematic flow diagram is created based on the dynamic process of the spread of COVID-19 infection in Figure 1. In addition, considering the transmission characteristics of COVID-19, relevant population quarantine measures, suspected cases (part of them are influenza patients or common pneumonia patients, etc.), three classifications of confirmed patients, the difference between clinically diagnosed cases and confirmed cases in Wuhan, and the lockdown of Wuhan and Hubei, we constructed the following differential equations to describe the COVID-19 model:

(4) $$\begin{array}{c} \left\{\begin{array}{c} \frac{dS}{dt}=-\frac{\beta_{1}S\left(pE+L+qI+kH\right)}{N}-\frac{\beta_{2}S\left(pE+L+qI\right)}{N}+\omega \eta L,\\ \frac{dE}{dt}=\frac{\beta_{1}S\left(pE+L+qI+kH\right)}{N}+\frac{\beta_{2}S\left(pE+L+qI\right)}{N}-\alpha E,\\ \frac{dL}{dt}=\alpha \rho E+\theta_{4}\sigma I-\left(\omega +\xi \right)L,\\ \frac{dI}{dt}=\alpha \left(1-\rho \right)E+\omega \left(1-\eta \right)L-\sigma I-dI,\\ \frac{{dH}_{1}}{dt}=\theta_{1}\sigma I-\delta_{1}H_{1}-d_{1}H_{1},\\ \frac{{dH}_{2}}{dt}=\theta_{2}\sigma I+\delta_{1}\lambda_{1}H_{1}-\delta_{2}H_{2}-d_{2}H_{2},\\ \frac{{dH}_{3}}{dt}=\theta_{3}\sigma I+\delta_{2}\lambda_{2}H_{2}-\delta_{3}H_{3}-d_{3}H_{3},\\ \frac{dR}{dt}=\left(1-\overset{4}{\underset{i=1}{\sum }}\theta_{i}\right)\sigma I+\delta_{1}\left(1-\lambda_{1}\right)H_{1}+\delta_{2}\left(1-\lambda_{2}\right)H_{2}+\delta_{3}H_{3}. \end{array}\right. \end{array}$$

### 2.2. The Basic Reproduction Number

The basic reproduction number (*ℛ*_0_) is the expected number of secondary cases produced, in a completely susceptible population, by a typical infective individual [51]. For threshold systems, if *ℛ*_0_ < 1, it means that the infection cannot grow and the disease will die out. Conversely, if *ℛ*_0_ > 1, then each infected individual produces more than one new infection on average, and the disease will become epidemic [52]. Of course, for nonthreshold systems, *ℛ*_0_ is no longer the only measure of whether a disease is extinct. However, a smaller value of *ℛ*_0_ can be advantageous to the control of the disease.

We define the following: *A* = (*ω* + *ξ*)(*σ* + *d*) − *θ*_4_*σ*(1 − *η*), *B* = *ρω*(1 − *η*) + (*ω* + *ξ*)(1 − *ρ*), *C* = (*δ*_1_ + *d*_1_)*A*, *G* = (*δ*_2_ + *d*_2_)*C*, *J* = (*δ*_3_ + *d*_3_)*G*, and *K* = *θ*_1_*δ*_1_*λ*_1_ + *θ*_2_(*δ*_1_ + *d*_1_).

By referring to the paper of Driessche and Watmough [33], *ℛ*_0_^(*i*)^, *i* = 1, 2 is calculated through the ordinary differential equations as follows:

(5) $$\begin{array}{c} {R_{0}}^{\left(1\right)}=R_{11}+R_{12}+R_{13}+R_{14}+R_{15}+R_{16}, \end{array}$$

where

(6) $$\begin{array}{c} \begin{array}{c} \begin{array}{c} R_{11}=\beta_{1}p/\alpha ,\\ R_{12}=\beta_{1}\left(\rho \left(\sigma +d\right)\theta_{4}\sigma \left(1-\rho \right)\right)/A,\\ R_{13}=\beta_{1}qB/A, \end{array}\\ \begin{array}{c} R_{14}=\beta_{1}k\theta_{1}\sigma B/C,\\ R_{15}=\beta_{1}k\left(\omega \rho \left(1-\eta \right)\sigma K+\sigma \left(\omega +\eta \right)\left(1-\rho \right)K\right)/G,\\ R_{16}=\beta_{1}kB\left(\left(\theta_{1}\delta_{1}\lambda_{1}+\theta_{2}\left(\delta_{1}+d_{1}\right)\right){\sigma \delta }_{2}\lambda_{2}+\theta_{3}\sigma \left(\delta_{1}+d_{1}\right)\left(\delta_{2}+d_{2}\right)\right)/J, \end{array} \end{array} \end{array}$$

(7) $$\begin{array}{c} {R_{0}}^{\left(2\right)}=R_{21}+R_{22}+R_{23}, \end{array}$$

where

(8) $$\begin{array}{c} \begin{array}{c} R_{21}=\beta_{2}p/\alpha ,\\ R_{22}=\beta_{2}\left(\rho \left(\sigma +d\right)+\theta_{4}\sigma \left(1-\rho \right)\right)/A,\\ R_{23}=\beta_{2}qB/A. \end{array} \end{array}$$

*ℛ*_0_^(1)^ represents the basic reproduction number of COVID-19, and the *ℛ*_0_^(2)^ represents the basic reproduction number of influenza or common pneumonia, etc.

### 2.3. Data Fitting and Parameter Estimation

#### 2.3.1. Data

The surveillance data of COVID-19 in Hubei province was reported by the Health Commission of Hubei Province (HCHP) [53] and WHO [54]. HCHP conducted daily briefings about COVID-19 data in all cities of Hubei province. Data information includes the cumulative number of reported cases (i.e., confirmed cases or confirmed cases plus clinical diagnosis cases), recovered cases, death cases, severe, and critical illness. The cumulative reported cases, severe cases, and critical cases data are from 10 January to 30 April 2020, a total of 112 days, and the death cases are from 10 January to 15 April 2020, a total of 97 days. The COVID-19 outbreak began in late January and was stabilized in April, which took nearly three months in Hubei.

#### 2.3.2. Parameter Estimation

We have determined the range of the transmission rate (*β*_*i*_, *i* = 1, 2) based on Abbott et al. [36] and Li et al. [23]. Referring to the results by Tang et al. [25], we set the corresponding range for mortality rate (*d*, *d*_1_, *d*_2_, and *d*_3_) and the proportion of infectious (*ρ*). The incubation period of the disease (1/*α*) is 5.1 days [55], so the transfer rate (from exposed individuals (*E*) to suspected patients (*L*) and clinically diagnosed cases (*I*)) is *α* = 0.1961. We referred to the values of *p*, *q*, and *k* in Chowell et al.'s result [56] and obtained the appropriate ranges of *p*, *q*, and *k*. We considered the classification of different types of patients: mild, severe, and critical. And we also collected daily cumulative reported cases, severe cases, and critical cases from HCHP, calculated the proportions of the three types of patients in the confirmed cases, and further determined the value range of *θ*_1_, *θ*_2_, *θ*_3_, and *θ*_4_ (for details, see Table 2). According to the Statistical Communique on Hubei province's National Economic and Social Development [57], we made assumptions about the range of the initial population of susceptible persons (*S*(0)). According to data related to COVID-19 reported by HCHP [34], *H*_2_(0) = 7, *R*(0) = 4, and *D*(0) = 1, we set the range of *H*_1_(0) and *H*_3_(0). Table 2 sets the upper and lower bounds of other parameters required by the model.

Here, we will introduce the simulation process in detail:

*(1) Before Wuhan Was Locked Down (10 January-22 January 2020)*. During this period of time, many people have no sense of protection against COVID-19, and the phenomenon of human-to-human transmission has not been confirmed. The transmission rate (*β*_1_) and the infection rate of medical staff (*k*) are both high.

*(2) After Wuhan Was Locked Down and before Hubei Was Locked Down (23 January-27 January 2020)*. Wuhan was locked down at 10:00 a.m. on 23 January 2020, and the last city in Hubei (Xiangyang) was locked down at 0:00 a.m. on 27 January 2020. With the gradual adoption of lockdown measures across the province, the transmission rate (*β*_1_) will be reduced to a certain extent, but there will be a certain degree of shortage of medical supplies.

*(3) After Hubei Was Locked Down and before the Large-Scale Case Screening (28 January-11 February 2020)*. On 5 February 2020, the National Health Commission (NHC) issued the “Diagnosis and Treatment Protocol for COVID-19 (Trial Version 5)” [7]. At the time of lockdown in Hubei, due to the shortage of medical materials such as nucleic acid test kits, Hubei had adopted a different diagnosis method from other provinces, adding “clinically diagnosed cases” [58]. Other provinces still divided cases into “suspected cases” and “confirmed cases.” With the complete lockdown in Hubei, the transmission rate (*β*_1_) further decreased. With the further shortage of nucleic acid testing kits and medical supplies, the time required for nucleic acid testing (1/*σ*) increased, and the risk of infection between medical personnel and confirmed patients (*k*) increased.

*(4) After Adding Clinically Diagnosed Cases to Cumulative Reported Cases and before Removing Clinically Diagnosed Cases from Cumulative Reported Cases (12 February-19 February 2020)*. On 12 February 2020, NHC added clinically diagnosed cases to the cumulative cases. The cumulative number of reported cases in Hubei province increased to 13,332 on that day. And on 19 February 2020, clinically diagnosed cases had been excluded from the cumulative cases. Here, we take the same *β*, *k*, and *q* as the third paragraph (After Hubei Was Locked Down and before the Large-Scale Case Screening (28 January-11 February 2020)).

Hence, the following equations could describe the dynamics of the cumulative reported cases (*Y*) in Hubei province:

(9) $$\begin{array}{c} \frac{dY}{dt}=\left\{\begin{array}{c} \left(\theta_{1}+\theta_{2}+\theta_{3}\right)\sigma I+\delta_{1}\lambda_{1}H_{1}+\delta_{2}\lambda_{2}H_{2}+\alpha \left(1-\rho \right)E+\omega \left(1-\eta \right)L,12\text{ February}‐19\text{ February }2020,\\ \left(\theta_{1}+\theta_{2}+\theta_{3}\right)\sigma I+\delta_{1}\lambda_{1}H_{1}+\delta_{2}\lambda_{2}H_{2},\text{other time}. \end{array}\right.\; \end{array}$$

*(5) After the Large-Scale Case Screening (after 19 February 2020)*. With the improvement of nucleic acid detection kits and medical conditions, the screening speed of clinically diagnosed cases had been further improved, and the previous cumulative clinically diagnosed cases were screened on 19 February 2020, so the NHC decided to remove clinically diagnosed cases from the cumulative cases. Here, we take the same *β*, *k*, and *q* as the third paragraph (After Hubei Was Locked Down and before the Large-Scale Case Screening (28 January-11 February 2020)).

To better fit the surveillance data, we divided the data into three stages from 10 January 2020 to 30 April 2020. These three stages set different transmission rates (*β*_*i*_, *i* = 1, 2), nucleic acid detection times (*σ*), the infection reduction factors between susceptible and confirmed cases (*k*), and the infection reduction factors between susceptible and clinically diagnosed cases (*q*). Different numbers of people leaving Hubei province are set on 23 and 27 January 2020 (i.e., the beginning and end of the second stage), and the rest parameters remain unchanged for the three stages.

The number of the cumulative reported cases in the early stage of the outbreak of COVID-19 is small, but the number in the mid-term is relatively large. In the later stage, it gradually stabilizes; hence, the numerical curve fluctuates greatly. We fit four sets of data at the same time, and each set of data has fluctuation. The Chi-square value can make the error of small data as small as possible, and the error of relatively large data is allowed to be larger, which can improve the fitting effect of the model. So, the Chi-square value is selected to evaluate the reliability of the model [22], and 35 parameters and 6 initial values are estimated by the fminsearch optimization method [59, 60].

(10) $$\begin{array}{c} J=\overset{112}{\underset{i=1}{\sum }}\frac{{\left(Y\left(t_{i}\right)-\overset{\wedge }{Y}\left(t_{i}\right)\right)}^{2}}{\hat{Y}\left(t_{i}\right)}+\overset{97}{\underset{i=1}{\sum }}\frac{{\left(D\left(t_{i}\right)-\overset{\wedge }{D}\left(t_{i}\right)\right)}^{2}}{\hat{D}\left(t_{i}\right)}+\overset{112}{\underset{i=1}{\sum }}\frac{{\left(H_{2}\left(t_{i}\right)-{\overset{\wedge }{H}}_{2}\left(t_{i}\right)\right)}^{2}}{{\hat{H}}_{2}\left(t_{i}\right)}+\overset{112}{\underset{i=1}{\sum }}\frac{{\left(H_{3}\left(t_{i}\right)-{\overset{\wedge }{H}}_{3}\left(t_{i}\right)\right)}^{2}}{{\hat{H}}_{3}\left(t_{i}\right)}. \end{array}$$

Here *Y*(*t*_*i*_), *i* = 1, 2, ⋯, 112 represents the actual cumulative reported cases per day, and $\hat{Y}\left(t_{i}\right),i=1,2,\cdots ,112$ represents the corresponding fitted values. *D*(*t*_*i*_), *i* = 1, 2, ⋯, 97 represents the actual number of deaths per day, and $\hat{D}\left(t_{i}\right),i=1,2,\cdots ,97$ represents the corresponding fitted values. *H*_2_(*t*_*i*_), *i* = 1, 2, ⋯, 112 represents the actual number of severely ill patients per day, and ${\hat{H}}_{2}\left(t_{i}\right),i=1,2,\cdots ,112$ represents the corresponding fitted values. *H*_3_(*t*_*i*_), *i* = 1, 2, ⋯, 112 represents the actual number of critically ill patients per day, ${\hat{H}}_{3}\left(t_{i}\right),i=1,2,\cdots ,112$ represents the corresponding fitted values, and *J* represents the total Chi-square value. Table 2 shows the parameter values and initial values of the model at different stages.

## 3. Results

In this section, we first give the simulation comparison results, then calculate *ℛ*_0_ for three different stages according to the parameter values fitted by the model. Secondly, since *ℛ*_0_ is a key threshold for measuring the level of epidemic transmission, the sensitivity of each parameter in *ℛ*_0_ is also worthy of our attention. Therefore, we will conduct a sensitivity analysis on *ℛ*_0_ to find parameters that have a greater impact on *ℛ*_0_.

### 3.1. The Result of Model-Based Estimates

**Figure 2:**
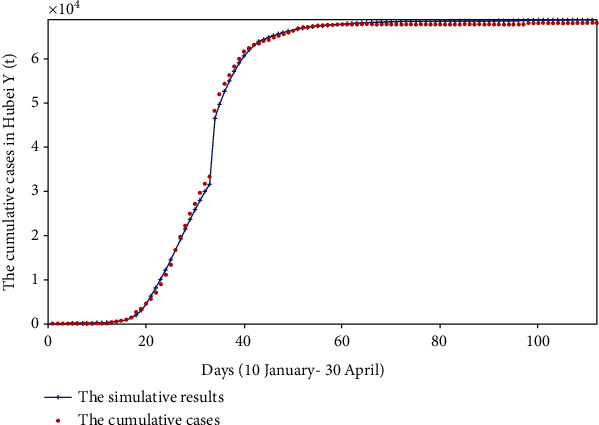
The cumulative reported and simulative cases in Hubei province (*Y*(*t*)).

**Figure 3:**
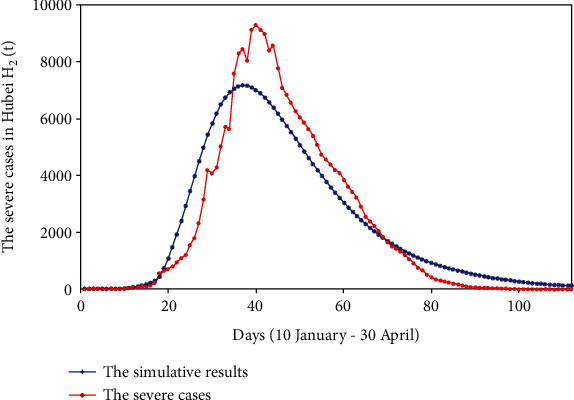
The severe cases and simulative cases in Hubei province (*H*_2_(*t*)).

**Figure 4:**
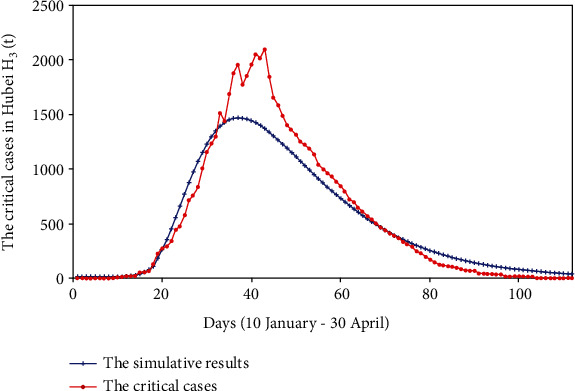
The critical cases and simulative cases in Hubei province (*H*_3_(*t*)).

**Figure 5:**
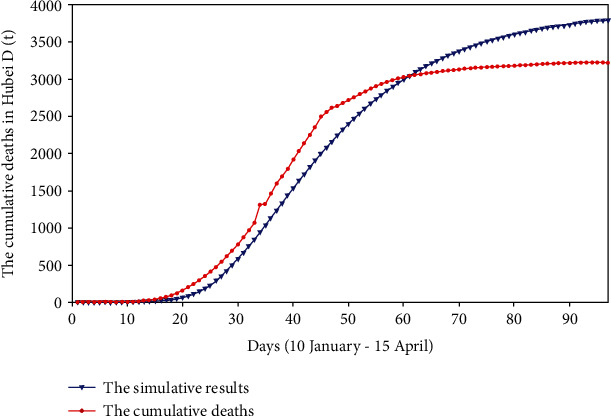
The death cases and simulative cases in Hubei province (*D*(*t*)).

We used the surveillance data found from the HCHP, the initial values, and the range of each parameter to perform the fminsearch optimization method. On this basis, the values of each parameter were continuously adjusted until the computer fit a suitable result. The fitting results of cumulative reported, severe cases, critical cases, and death cases are shown in Figures 2–5.

We fit four sets of data at the same time; from Figures 2–5, we can see that the cumulative reported cases fit the best, and the other three sets of data can still fit the basic trend, because our model takes multiple actual factors into consideration, including suspected cases (part of them are influenza patients or common pneumonia patients, etc.), quarantine, patient classification (three types), clinically diagnosed cases, and lockdown of Wuhan and Hubei. The above considerations lead to high dimensions and many parameters of the model, so we need to fit multiple sets of data at the same time to verify the rationality of the model parameters. Therefore, the fitting result is obtained by cross-validation of the four sets of data.

### 3.2. Results of Basic Reproduction Numbers

The results of basic reproduction numbers at the three different stages will be described in this part. From the COVID-19 model, we got that when Wuhan was not locked down (10 January-22 January), *ℛ*_0_ = 3.1571 in Hubei province; after Wuhan was locked down but Hubei was not locked down (23 January-27 January), *ℛ*_0_ = 2.0471; after Hubei was locked down (28 January-30 April), *ℛ*_0_ = 1.5014.

With the implementation of various control measures, the value of the basic reproduction number is constantly decreasing, which shows that timely lockdown, patient classification, and large-scale case screening are a series of effective control measures.

### 3.3. Sensitivity Analysis of Basic Reproduction Numbers

Sensitivity analysis of the basic reproduction number is performed in this section to determine several parameters that have the greatest influence on the prevalence and transmission of COVID-19. Through the sensitivity analysis of the basic reproduction number *ℛ*_0_, we can find the most sensitive parameter to *ℛ*_0_. Then, we can reduce the basic reproduction number by making relevant measures to control the parameters and further achieve the purpose of disease control.

According to the research method of Samsuzzoha et al. [61] on the sensitivity analysis of the basic reproduction number, we used the simulated parameter values (the third stage, i.e., after 28 January 2020) to perform a sensitivity analysis of the basic reproduction number. If a small change in a parameter can cause a large change in the value of the basic reproduction number, then this parameter is called a sensitivity factor, otherwise called an insensitive factor. The sensitivity indices of each parameter about the basic reproduction number *ℛ*_0_ are shown in Table 3 (by increasing the value of the basic reproduction number *ℛ*_0_ by 1%, observe the corresponding changes in the sensitivity index of the parameter).

From Table 3, we can observe that *β*_2*c*_, *p*, *q*_*c*_, *θ*_4_, *ω*, *k*_*c*_, *θ*_1_, *θ*_2_, *λ*_2_, *λ*_1_, and *θ*_3_ have a positive impact on *ℛ*_0_. *ξ*, *ρ*, *α*, *σ*_*c*_, *η*, *δ*_1_, *δ*_2_, and *δ*_3_ have a negative impact on *ℛ*_0_. Sensitivity analysis shows that the basic reproduction number is highly sensitive to *β*_2*c*_, *p*, *ξ*, *ρ* and *α*. Therefore, the lower transmission rate (*β*), the lower infectiousness reduction factor (*p*), and the lower proportion of the infectious (1 − *ρ*) can effectively reduce the basic reproduction number.

From the sensitivity analysis of parameters, we can see that *k*, *q*, and *σ* are not very sensitive which shows that their valuations have certain randomness and complexity. The parameters do not present in monotonous changes like increasing or decreasing. This situation appeared to show the complexity of the epidemic through transmission and the comprehensive impact of the number of patients in each stage, the limited degree of medical resources, the development of nucleic acid testing kits, and so on. So, when we use this model to describe the phenomenon, it can also reflect the complexity of the epidemic.

## 4. Discussion

With the implementation of various control measures, the basic reproduction number was decreasing at different stages. But we do not know how much positive effect is brought by patient classification and lockdown. Next, we will discuss the following three situations in detail to introduce the impact on Hubei from 10 January to 18 April.

### 4.1. The Impact of Patient Classification on COVID-19

Assuming that patients are not classified, we modify the model. In this new model, we merge the previous *H*_1_, *H*_2_, and *H*_3_ into *H*, change the sum of *θ*_1_, *θ*_2_, and *θ*_3_ in the previous model to *θ*_1_ = 0.3443 in the current model, and change *θ*_4_ in the previous model to *θ*_2_ = 0.4870 in the current model, then modify *δ*_2_ in the previous model to *δ* = 0.0593 in the current model. The model is described by the following ordinary differential equations:

(11) $$\begin{array}{c} \left\{\begin{array}{c} \frac{dS}{dt}=-\frac{\beta_{1}S\left(pE+L+qI+kH\right)}{N}-\frac{\beta_{2}S\left(pE+L+qI\right)}{N}+\omega \eta L,\\ \frac{dE}{dt}=\frac{\beta_{1}S\left(pE+L+qI+kH\right)}{N}+\frac{\beta_{2}S\left(pE+L+qI\right)}{N}-\alpha E,\\ \frac{dL}{dt}=\alpha \rho E+\theta_{2}\sigma I-\left(\omega +\xi \right)L,\\ \frac{dI}{dt}=\alpha \left(1-\rho \right)E+\omega \left(1-\eta \right)L-\sigma I-dI,\\ \frac{dH}{dt}=\theta_{1}\sigma I-\delta H-d_{1}H,\\ \frac{dR}{dt}=\left(1-\theta_{1}-\theta_{2}\right)\sigma I+\delta H. \end{array}\right. \end{array}$$

After the patient is hospitalized, the hospital will adopt different treatment plans according to the different symptoms of the patients, which is equivalent to classifying the patients [7], so we discuss the situation of not classifying the case of the patients. In this case, we assume that the hospital does not classify and isolate clinically diagnosed cases according to the severity of pneumonia imaging features; in other words, the hospital treats all clinically diagnosed cases equally, and everyone will receive the subsequent nucleic acid test with equal probability. It will increase the probability of contact between COVID-19 carriers and non-COVID-19 carriers in clinically diagnosed cases and further increase the probability of infection between non-COVID-19 carriers.

**Figure 6:**
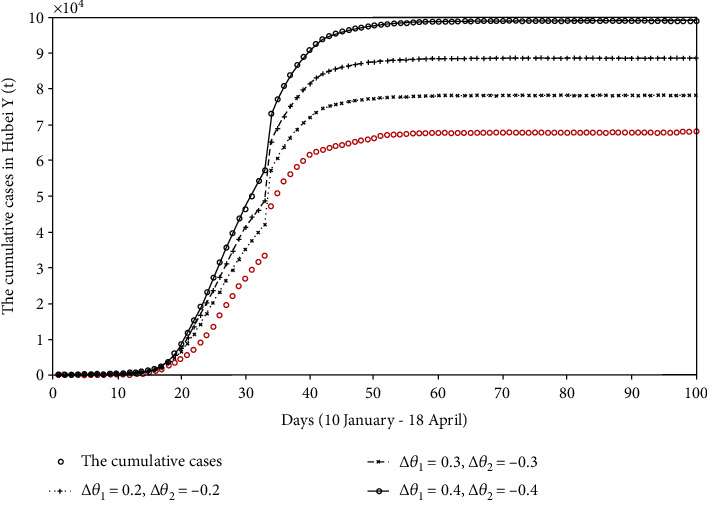
The comparison chart of the cumulative reported cases if the patient is not classified.

This series of reactions reflected in the model will lead *θ*_1_ to a further increase, and the corresponding *θ*_2_ will decrease to a certain extent. Therefore, we propose Δ*θ*_*i*_(*i* = 1, 2) to describe the change of scale factor (*θ*). If Δ*θ* > 0, it means that the value of *θ* will increase compared to the fitted value. Conversely, if Δ*θ* < 0, the value of *θ* will be reduced. The final cumulative reported cases are shown in Figure 6.

In Figure 6, we can see that if the patients are not classified, the final disease scale will increase. If *θ*_1_ = 0.5443 and *θ*_2_ = 0.2870 (Δ*θ*_1_ = 0.2, Δ*θ*_2_ = −0.2), the cumulative number of reported cases will reach 7.8255 × 10^4^. If *θ*_1_ = 0.7443 and *θ*_2_ = 0.0870 (Δ*θ*_1_ = 0.4, Δ*θ*_2_ = −0.4), the final scale of the disease will reach 9.9087 × 10^4^, which is 1.45 times that of the actual cumulative number of reported cases.

### 4.2. The Impact of Lockdown on COVID-19

In this part, we will consider the impact of lockdown on the prevention and control of the epidemic in Hubei. In Figure 1, the suspected patients who were introduced into compartment *L* will be taken to three different compartments after case screening. Among them, people from compartment *L* to compartment *I* represent a part of the suspected patients who have been identified as confirmed patients and should be hospitalized for isolation; in addition, people from compartment *L* to compartment *C* are the suspected patients who are not COVID-19 carriers, so they will be in strict home quarantine, and the probability that they will be infected in the short term will be ignored. In that case, they should be removed from the system. People from compartment *L* to compartment *S* denote that these suspected patients do not carry COVID-19, but they cannot be quarantined at home for some reasons, such as being a volunteer or medical staff. These people will continue to stay in the system. Therefore, when we do not take measures to lock down the city, we consider two situations from compartment *L* to compartment *C*. In the first case, we still remove the people who are in strict home quarantine from the system; in another case, these people are not removed from the system and are allowed to continue to stay in the system.

#### 4.2.1. Supposed Wuhan Was Locked Down, Hubei Was Not Locked Down

**Figure 7:**
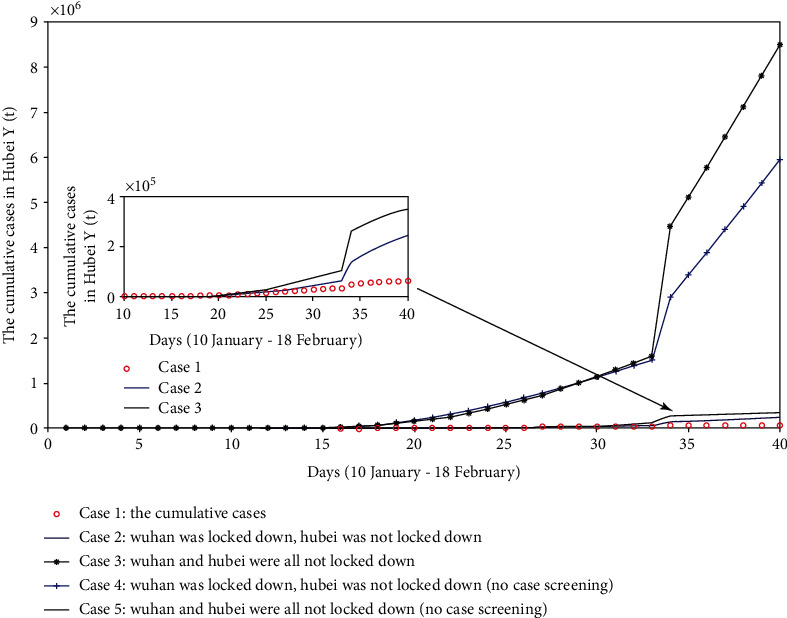
The comparison chart of cumulative reported cases when discussing whether Wuhan and Hubei are locked down or not.

Here, we postulate that Wuhan was locked down on 23 January 2020, but Hubei province did not do a lockdown to control population movements. So, we use the parameters of the first stage (before Wuhan was locked down, i.e., 10 January-22 January 2020) and the second stage (after Wuhan was locked down and before Hubei was locked down, i.e., 23 January-27 January 2020) to simulate the cumulative reported cases and use the values of relevant parameters *φ*_*a*_ in the second stage to simulate the population movement before the lockdown of Wuhan. The fitting results are shown in Figure 7.

#### 4.2.2. Supposed Wuhan and Hubei Were Not Locked Down

From 10 January to 30 April, we assume that the entire Wuhan and Hubei provinces did not take a lockdown measure to control population movements. The parameter values of the first stage (before Wuhan was locked down, i.e., 10 January-22 January 2020) are used to simulate the cumulative reported cases, and the fitting results are shown in Figure 7.

From Figure 7, when we do not remove the compartment *C*, we can see that the scale of the epidemic will reach 2.4677 × 10^5^ on 18 February 2020, if Wuhan was locked down but Hubei was not locked down. But, if the suspected patients who do not carry COVID-19 after case screening are not quarantined at home, they will be the susceptible people again and still be in the system (the case of removing compartment *C*). At this time, if Wuhan and Hubei do not impose a lockdown measure, the confirmed cases will rise by 8.5097 × 10^6^ on 18 February 2020, which is 138 times that of the actual number of reported cases.

### 4.3. The Impact of the Lockdown Time on COVID-19

According to the previous fitting results, after the lockdown of Wuhan, we removed 8.8731 × 10^6^ people (*φ*_*a*_) from the system's susceptible compartment; after the lockdown of Hubei, we removed 1.5910 × 10^7^ people (*φ*_*b*_) from the system's susceptible compartment. Here, we introduce Δ*φ* to characterize the change in the number of susceptible caused by the change of the lockdown time.

If Δ*φ* > 0, it means that the number of susceptible individuals has increased compared to the previous fitted value. If Δ*φ* < 0, it means that the number of susceptible individuals is reduced compared with the previous fitted value.

(12) $$\begin{array}{c} \Delta \varphi =\left\{\begin{array}{c} \Delta \varphi_{a},\text{with the change of lockdown time of Wuhan},\text{the change value},\\ \Delta \varphi_{b},\text{with the change of lockdown time of Hubei},\text{the change value}. \end{array}\right. \end{array}$$

#### 4.3.1. Wuhan Lockdown Time Remains Unchanged

**Figure 8:**
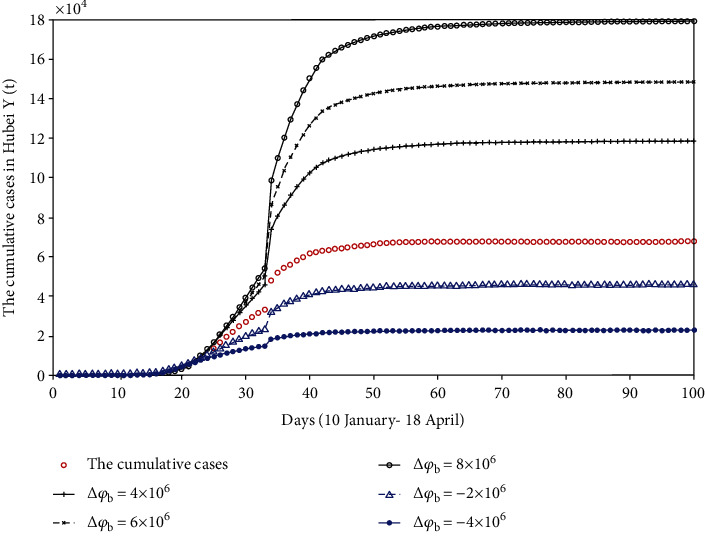
The comparison chart of cumulative reported cases when Wuhan lockdown time remains unchanged, but Hubei lockdown time changed.

*(1) The Lockdown Time of Hubei Was Delayed*. The lockdown time of Xiangyang city is set as the lockdown time of Hubei province because Xiangyang city is the last city in Hubei province to be locked down. Here, we assume that the lockdown time of Hubei province is 30 January. Compared with the previous lockdown of Hubei on 27 January, during the three days from 27 January to 30 January, some people will not be able to stay in quarantine. Therefore, these people are added to the susceptible (number of susceptible on the 21st day, *S*(21)). Then, we discuss the impact of the different number of susceptible patients on the final disease scale. The fitting results are shown in Figure 8.

*(2) The Lockdown Time of Hubei Was Advanced*. Based on the lockdown of Wuhan on 23 January, we assume that the lockdown time of Hubei province is two days earlier than the actual lockdown time. That is to say, Hubei province completed the lockdown on 25 January. Due to the advance of the lockdown, some people should stay in quarantine. We get rid of these people from the susceptible (*S*(16)) and discuss the impact of the different number of susceptible patients on the final disease scale. The fitting results are shown in Figure 8.

From Figure 8, if Hubei province completes the lockdown on 30 January, the final scale of the disease will increase, and when the number of susceptible people increases by 8 million, the final scale of the disease will be controlled at 1.7934 × 10^5^. We can also see that if Hubei province completes the lockdown on 25 January, the final scale of the disease will become small and when the number of susceptible people is reduced by 4 million, the final disease scale will be controlled at 2.3002 × 10^4^. Therefore, when the lockdown time in Wuhan is fixed, the advancement of the lockdown time in Hubei has a positive effect on disease control.

#### 4.3.2. Wuhan and Hubei Are All Locked Down in Advance or Delayed

**Figure 9:**
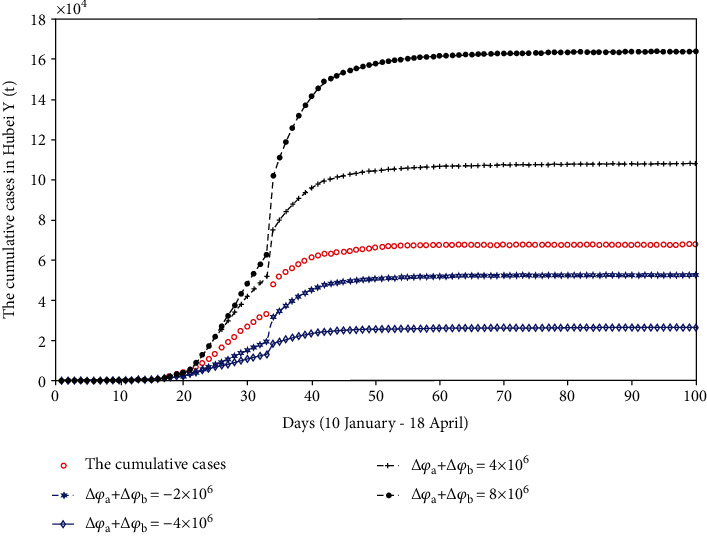
The comparison chart of cumulative reported cases when Wuhan and Hubei are all locked down in advance or delayed.

*(1) Wuhan Lockdown Time Was Advanced, and Hubei Lockdown Time Was also Advanced*. In this part, we assume that Wuhan city was locked down on 20 January 2020, and Hubei was locked down on 23 January 2020. Compared with the actual situation, with the advancement of lockdown time, the population flow during the Spring Festival will decrease, which will further reduce the number of susceptible people. We get rid of these people from the susceptible (*S*(11) and *S*(14)). For these people, we discuss the impact of different numbers on the final disease scale. The fitting results are shown in Figure 9.

*(2) The Lockdown Time of Wuhan and Hubei Were Both Delayed*. Supposing Wuhan was locked down on 25 January 2020, and Hubei was locked down on 30 January 2020. With the postponement of the lockdown time, the scale of population flow will expand, leading to more susceptible people than the actual situation. Thus, they have been added to the susceptible (*S*(16) and *S*(21)). The fitting results are shown in Figure 9.

If both Wuhan and Hubei are locked down in advance, the final scale of the disease will be well controlled, and when the number of susceptible people is reduced by 4 million, the final disease scale will be controlled at 2.6501 × 10^4^. On the contrary, if the lockdown time of Wuhan and Hubei are delayed, the final scale of the disease will increase, and when the number of susceptible people increases by 8 million, the final scale of the disease will be up to 1.6393 × 10^5^, which is 2.41 times that of the actual number of reported cases.

#### 4.3.3. The Lockdown Time of Wuhan and Hubei in which One Is Advanced and Another Is Delayed

**Figure 10:**
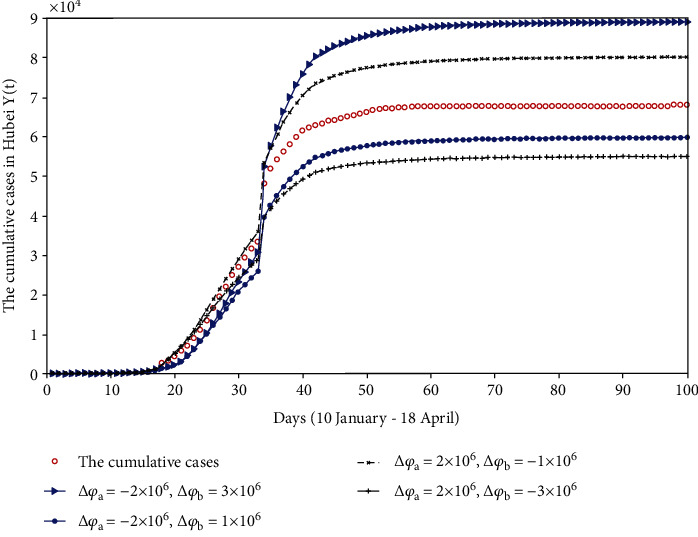
When the lockdown time of Wuhan and Hubei in which one is advanced and the other is delayed, the comparison chart of cumulative reported cases.

*(1) Wuhan Lockdown Time Was Advanced, and Hubei Lockdown Time Was Delayed*. We assumed that Wuhan was locked down on 20 January 2020, and Hubei was locked down on 30 January 2020 in this situation. With the early lockdown of Wuhan, we get rid of these people (Δ*φ*_*a*_ = −2 × 10^6^) from the susceptible (*S*(11)), but the delay for the lockdown of Hubei will make more and more people become susceptible, so we added these people to the susceptible (*S*(21)). The fitting results are shown in Figure 10.

*(2) Wuhan Lockdown Time Was Delayed, and Hubei Lockdown Time Was Advanced*. Presuming that Wuhan was locked down on 24 January 2020, and Hubei was locked down on 26 January 2020. With the postponement of Wuhan's lockdown, some people will go out and become susceptible, and we add these people (Δ*φ*_*a*_ = 2 × 10^6^) to the susceptible (*S*(15)), but the advancement of the time for the lockdown of Hubei will cause a number of people to quarantine at home, which will decrease the number of susceptible people, so we remove these people from susceptible (*S*(17)). The fitting results are shown in Figure 10.

From Figure 10, we can see that the scale of the disease is closely related to the change in the number of susceptible people. When Wuhan was locked down in advance (Δ*φ*_*a*_ = −2 × 10^6^), we made two different assumptions about the number of susceptible people caused by the delay in Hubei's lockdown. If Δ*φ*_*b*_ = 3 × 10^6^, the cumulative cases will be up to 8.9039 × 10^4^; if Δ*φ*_*b*_ = 1 × 10^6^, the final scale of the disease will be controlled at 5.9814 × 10^4^. When Wuhan postponed the lockdown time of the city (Δ*φ*_*a*_ = 2 × 10^6^), we also made two different assumptions. If Δ*φ*_*b*_ = −1 × 10^6^, the cumulative number of confirmed cases of the disease will reach 8.0203 × 10^4^; if Δ*φ*_*b*_ = −3 × 10^6^, the final scale of the disease will be reduced to 5.5057 × 10^4^. Through our multiple tests, if Δ*φ*_*a*_ + Δ*φ*_*b*_ < 0, the cumulative cases will be less than the actual cases; if Δ*φ*_*a*_ + Δ*φ*_*b*_ > 0, the number of the cumulative cases will be more than that of the actual cases.

### 4.4. Data Revision and Comparative Analysis

By referring to the results of Lipsitch et al. [62], we use an exponential function to revise the sudden increase of the number of confirmed cases on 12 February 2020 and allocate more than 10,000 people to the days from 10 January to 12 February. We assume that the number of daily new cases after 12 February is the real data, so we take *t* = 34 from the following formula.

(13) $$\begin{array}{c} \left\{\begin{array}{c} Y\left(t\right)=e^{\lambda t},\\ \lambda =\ln\left(Y\left(34\right)\right)/34. \end{array}\right. \end{array}$$

**Figure 11:**
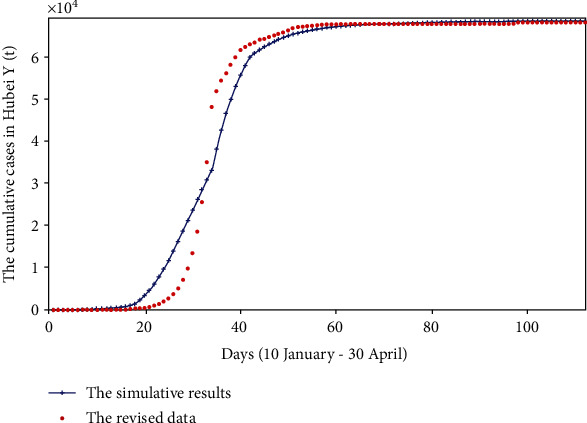
The revised cumulative reported and simulative cases in Hubei province (*Y*(*t*)).

Therefore, the revised cumulative number of confirmed cases from 10 January to 12 February can be obtained, and then, we can use the fminsearch optimization method to get Figure 11.

Comparing with Figure 2, by calculating the minimum sum of the Chi-square, we get that the minimum sum of Chi-square in Figure 2 is 2.44 × 10^3^, and the minimum sum of Chi-square in Figure 11 is 9.09 × 10^4^. Therefore, the model of this paper is more suitable for describing the real reported data and less suitable for fitting smoothed data.

## 5. Conclusion

COVID-19 has now become a global epidemic, which has had a huge impact on all aspects of human beings. The outbreak broke out in Wuhan at the end of 2019 and the epidemic in Hubei had been fully controlled as early as late April 2020. The next time, although some small-scale epidemics still occurred in Beijing [63], Xinjiang [64], Liaoning [65], Hebei [66], Heilongjiang [67], Jilin [68], Jiangsu [69], Henan [70], Fujian [71], Inner Mongolia [72], and other places one after another, the cases can be cleared very quickly by taking measures like timely lockdown, patient classification, and large-scale case screening, which indicates that these actions are effective to control the epidemic. Obviously, the burden of treatment after the outbreak is far greater than the burden of disease prevention and control. Globally, as of 6:11 p.m. CET, 9 March 2022, the global cumulative confirmed cases number has exceeded 440 million, and the death cases number has exceeded 6 million [54]. In the meantime, the epidemic in foreign countries and other regions is still quite serious.

In this study, taking into account the five factors of suspected cases, quarantine, lockdown of Wuhan and Hubei, patient classification, and clinically diagnosed cases, we built a COVID-19 model to simulate the spread of COVID-19 in Hubei.

Firstly, we collected surveillance data published by the Hubei Provincial Health Commission and used the fminsearch optimization method to find the optimal parameter values. Next, the basic reproduction number from 10 January to 30 April (three stages) was calculated using the above parameter values. Before Wuhan was locked down (10 January-22 January), *ℛ*_0_ = 3.1571; after Wuhan was locked down but Hubei was not locked down (23 January-27 January), *ℛ*_0_ = 2.0471; after Hubei was locked down (28 January-30 April), *ℛ*_0_ = 1.5014. From the results of the basic reproduction number, we can find that the control measures taken by Hubei government can curb the spread of COVID-19 to a certain extent. Our results of *ℛ*_0_ are in agreement with other groups [12, 17, 19, 21, 28], and details can be seen in Table 1.

After that, we performed a sensitivity analysis on *ℛ*_0_, in which *β*, *p*, and (1 − *ρ*) were more sensitive than other parameters. Therefore, it can be concluded that strengthening personal protection [73], such as wearing masks, quarantining at home, and not going out unless necessary can effectively reduce the transmission rate (*β*). Keeping social distance and closing large entertainment venues [74] can reduce the infectivity reduction factor (*p*). Expanding the proportion and scope of nucleic acid testing, strengthening the test of close contacts and subclose contacts, and ensuring that all those in need are tested [2] can reduce the proportion of the infectious (1 − *ρ*).

Then, we discussed whether the patients were classified, whether Wuhan and Hubei were locked down, and the impact of the lockdown time adjustment on the final scale of the disease. Finally, we concluded that classifying and treating confirmed cases according to symptoms and locking down the city in Hubei as soon as possible can effectively reduce the number of cumulative confirmed cases, thereby further effectively curbing the spread of the epidemic. At present, the global epidemic is still very serious, and it is hoped that our research can provide some help for the epidemic prevention and give some references for other countries or regions.

Because the time period we studied was in the early stage of the epidemic in Wuhan, Hubei, some follow-up factors were not involved in our paper, such as asymptomatic infections [75], vaccines [76], imported cases [77], and coronavirus variants [78]. Considering asymptomatic infections, Serhani and Labbardi [79] build an *SIAQRD* model and take asymptomatic people, the isolation of an infected person, and the quarantine of contacting people into consideration. The result illustrates that the level of containment is of great importance. Thinking about the impact of imported cases, Boldog et al. [80] establish a time-dependent compartmental model to estimate the cumulative number of cases in China and use the Galton-Watson branching process to model the initial spread of COVID-19. Among that, they think about the factor of imported cases and incorporate it into the formula of risk estimation. They finally find that the actual connectivity and the local reproduction number have an effect on assessing the risks of major outbreaks from imported cases outside China.

In the future, we can further carry out follow-up research in these directions: sporadic cases brought by asymptomatic infected persons will lead to the local spread of the epidemic and the impact of vaccines and coronavirus variants on COVID-19.

## Figures and Tables

**Table 1:** The results of *ℛ*_0_ in Wuhan, Hubei or China.

Area	Research time	Value (95% or 90% CI)	Resources
China	Jan. 22, 2020-Mar. 30, 2020	0.454	Collins and Duffy [9]
Wuhan	Feb. 2, 2020-Feb. 11, 2020	0.84 (0.81-0.88)	Mizumoto et al. [11]
Wuhan	Jan. 4, 2020-Mar. 9, 2020	0.945	Ndaïrou et al. [29]
China	Jan. 24, 2020-Feb. 8, 2020	0.99 (0.76-1.33)	Li et al. [30]
Wuhan	Feb. 4, 2020-Feb. 12, 2020	1.08	Zhang et al. [21]
Wuhan	Jan. 23, 2020-Feb. 1, 2020	1.3065 (0.5273-2.0858)	Xue et al. [31]
Wuhan	Jan. 21, 2020-Feb. 8, 2020	1.32 (1.16-1.48)	Du et al. [32]
Wuhan	Jan. 15, 2020-Feb. 4, 2020	1.3469, 2.8349	Sun et al. [16]
China	Jan. 24, 2020-Feb. 3, 2020	1.36 (1.14-1.63)	Li et al. [30]
China	Jan. 18, 2020	1.40-2.50	WHO [33]
Wuhan	Jan. 23, 2020-Feb. 3, 2020	1.55	Zhang et al. [21]
Wuhan	Jan. 24, 2020-Feb. 11, 2020	1.7549	Pang et al. [17]
Wuhan	Jan. 25, 2020	2.00-3.00	Abbott et al. [27]
China	Jan. 24, 2020	2.10 (2.00-2.20)	Jung et al. [12]
China and overseas	Jan. 18, 2020	2.20 (90% HDI 1.40-3.80)	Riou and Althaus. [13]
Wuhan	Before Jan. 22, 2020	2.20 (1.40-3.90)	Li et al. [14]
Wuhan	Jan. 10, 2020-Jan. 24, 2020	2.24 (1.96-2.55)	Zhao et al. [15]
Wuhan	Jan. 10, 2020-Jan. 23, 2020	2.38 (2.03-2.77)	Li et al. [30]
Wuhan	Dec. 10, 2019-Jan. 21, 2020	2.42	Hu et al. [10]
Wuhan	Jan. 1, 2020-Jan. 15, 2020	2.56 (2.49-2.63)	Zhao et al. [34]
Wuhan	Jan. 23, 2020	2.57 (90% CI 2.37-2.78)	Chinazzi et al. [18]
China	Jan. 20, 2020-Feb. 11, 2020	2.68	Liu et al. [35]
Wuhan	Dec. 31, 2019-Jan. 25, 2020	2.68 (2.47-2.86)	Wu et al. [36]
Wuhan	Jan. 18, 2020-Feb. 13, 2020	2.70	Guo et al. [37]
Wuhan	Jan. 23, 2020-Mar. 6, 2020	2.71	Wang et al. [26]
Wuhan	Dec. 22, 2019-Mar. 15, 2020	2.80	Musa et al. [38]
China	Jan. 25, 2020	2.80-3.30	Zhou et al. [39]
Wuhan	Dec. 12, 2019-Feb. 22, 2020	3.04	Huang et al. [40]
Wuhan	Jan. 1, 2020-Jan. 22, 2020	3.11 (2.39-4.13)	Read et al. [28]
China	Dec. 31, 2019-Jan. 23, 2020	3.15 (3.04-3.26)	Tian et al. [19]
China	Jan. 24, 2020	3.20 (2.70-3.70)	Jung et al. [12]
Wuhan	Jan. 24, 2020-Feb. 5, 2020	3.30 (2.66-3.95)	Ma et al. [41]
China (excluding Hubei province)	Jan. 20, 2020-Mar. 3, 2020	3.36 (3.20-3.64)	Wan et al. [20]
Wuhan	Jan. 11, 2020-Jan. 23, 2020	3.4074 (2.9959-3.8188)	Xue et al. [31]
Wuhan	2019-2020	3.49 (3.39-3.62)	Mizumoto et al. [11]
Wuhan	Dec. 7, 2019-Jan. 1, 2020	3.58	Chen et al. [42]
Wuhan	Jan. 10, 2020-Jan. 24, 2020	3.58 (2.89-4.39)	Zhao et al. [15]
Wuhan	Dec. 8, 2019-Jan. 22, 2020	3.6	Zhang et al. [21]
Wuhan	Jan. 26, 2020-Feb. 9, 2020	3.66	Wang et al. [43]
Hubei	Jan. 27, 2020-Feb. 11, 2020	3.7732	Li et al. [22]
Wuhan	Jan. 10, 2020-Jan. 30, 2020	4.30	Song et al. [44]
Wuhan	Dec. 31, 2019-Jan. 23, 2020	4.6355	Pang et al. [17]
Hubei	Jan. 11, 2020-Jan. 22, 2020	5.6015	Li et al. [22]
Hubei	Jan. 23, 2020-Feb. 19, 2020	5.6870, 2.2426, 1.0560	Jia et al. [23]
Wuhan	Jan. 15, 2020-Jan. 30, 2020	5.70 (3.80-8.90)	Sanche et al. [45]
China	Jan. 10, 2020-Feb. 4, 2020	5.78 (5.71-5.89)	Wang et al. [46]
China	Jan. 10, 2020-Jan. 22, 2020	6.47 (5.71-7.23)	Tang et al. [24]
China	Jan. 23, 2020-Jan. 26, 2020	6.6037	Li et al. [22]
Wuhan	Jan. 4, 2020-Jan. 23, 2020	7.53	Song et al. [25]

**Table 2:** Parameter estimates for COVID-19 model in Hubei.

Parameter	Definitions	Value	Source
*β*_1*a*_ ∈ [0.1, 10]	Transmission rate (day^‐1^individual^‐1^)	1.8208	Estimated
*β*_2*a*_ ∈ [0.01, 1]	Transmission rate	0.5487	Estimated
*β*_1*b*_ ∈ [0.1, 10]	Transmission rate	1.1720	Estimated
*β*_2*b*_ ∈ [0.01, 1]	Transmission rate	0.3648	Estimated
*β*_1*c*_ ∈ [0.1, 10]	Transmission rate	0.8665	Estimated
*β*_2*c*_ ∈ [0.01, 1]	Transmission rate	0.5643	Estimated
*d* ∈ [0, 0.0001]	Mortality rate of clinically diagnosed cases (day^−1^)	3.8858 × 10^−5^	Estimated
*d*_1_ ∈ [0, 0.001]	Mortality rate of mild patients (day^−1^)	2.0436 × 10^−4^	Estimated
*d*_2_ ∈ [0, 0.2]	Mortality rate of severely ill patients (day^−1^)	4.5000 × 10^−4^	Estimated
*d*_3_ ∈ [0, 0.2]	Mortality rate of critically ill patients (day^−1^)	0.0641	Estimated
*δ*_1_ ∈ [0.1429, 0.5]	Recovery rate of mild patients (day^−1^)	0.2157	Estimated
*δ*_2_ ∈ [0.05, 0.5]	Recovery rate of severely ill patients (day^−1^)	0.0606	Estimated
*δ*_3_ ∈ [0.03, 0.2]	Recovery rate of critically ill patients (day^−1^)	0.0477	Estimated
*η* ∈ [0.01, 0.25]	Self-healing ratio	0.2117	Estimated
*k*_*a*_ ∈ [0.01, 0.4]	Infectivity reduction factor	0.0446	Estimated
*k*_*b*_ ∈ [0.01, 0.4]	Infectivity reduction factor	0.0259	Estimated
*k*_*c*_ ∈ [0.01, 0.4]	Infectivity reduction factor	0.0705	Estimated
*q*_*a*_ ∈ [0.1, 0.5]	Infectivity reduction factor	0.1561	Estimated
*q*_*b*_ ∈ [0.1, 0.5]	Infectivity reduction factor	0.2544	Estimated
*q*_*c*_ ∈ [0.1, 0.5]	Infectivity reduction factor	0.2282	Estimated
*ξ* ∈ [0.01, 1]	System population reduction factor	0.9700	Estimated
*λ*_1_ ∈ [0.1, 0.3]	Scale factor	0.3000	Estimated
*λ*_2_ ∈ [0.1, 0.6]	Scale factor	0.2499	Estimated
*ω* ∈ [0.016, 0.1]	Infectivity reduction factor	0.0177	Estimated
*p* ∈ [0.1, 0.5]	Infectivity reduction factor	0.1364	Estimated
*ρ* ∈ [0.1, 1]	Proportion of the infectious	0.9984	Estimated
*σ*_*a*_ ∈ [0.1, 0.5]	Nucleic acid detection time (day^−1^)	0.1472	Estimated
*σ*_*b*_ ∈ [0.1, 0.5]	Nucleic acid detection time	0.1309	Estimated
*σ*_*c*_ ∈ [0.1, 0.5]	Nucleic acid detection time	0.2576	Estimated
*θ*_1_ ∈ [0.2381, 0.3969]	Scale factor	0.2411	Estimated
*θ*_2_ ∈ [0.0471, 0.0786]	Scale factor	0.0786	Estimated
*θ*_3_ ∈ [0.0148, 0.0246]	Scale factor	0.0246	Estimated
*θ*_4_ ∈ [0.3, 0.55]	Scale factor	0.4870	Estimated
*φ*_*a*_ ∈ [0, 2 × 10^7^]	System discharges	8.8731 × 10^6^	Estimated
*φ*_*b*_ ∈ [1 × 10^7^, 5.907 × 10^7^]	System discharges	1.5910 × 10^7^	Estimated
*S*(0) ∈ [1 × 10^4^, 5.927 × 10^7^]	Initial susceptible population	3.1372 × 10^7^	Estimated
*E*(0) ∈ [2000, 1 × 10^7^]	Initial exposed population	2.2380 × 10^3^	Estimated
*L*(0) ∈ [0, 1 × 10^6^]	Initial suspected population	55.5580	Estimated
*I*(0) ∈ [0, 1 × 10^4^]	Initial clinically diagnosed cases	497.9463	Estimated
*H*_1_(0) ∈ [0, 41]	Initial mild patients	5.7737	Estimated
*H*_3_(0) ∈ [0, 41]	Initial critical patients	11.0952	Estimated

**Table 3:** The sensitivity indices of each parameter to the basic reproduction number.

Parameter	Sensitivity index to *ℛ*_0_	Percentage of corresponding change (%)
*β*_2*c*_	0.9996	−1.0004
*p*	0.4020	−2.4877
*q*_*c*_	0.0081	−1.2392 × 10^2^
*θ*_4_	0.0046	−2.1799 × 10^2^
*ω*	0.0017	−5.9956 × 10^2^
*k*_*c*_	0.0011	−9.3002 × 10^2^
*θ*_1_	8.2297 × 10^−4^	−1.2151 × 10^3^
*θ*_2_	4.6762 × 10^−4^	−2.1385 × 10^3^
*λ*_2_	2.1489 × 10^−4^	−4.6535 × 10^4^
*λ*_1_	1.0618 × 10^−4^	−9.4181 × 10^3^
*θ*_3_	1.4129 × 10^−5^	−7.0778 × 10^3^
*ξ*	−0.5904	1.6937
*ρ*	−0.4741	2.1093
*α*	−0.4020	2.4877
*σ*_*c*_	−0.0080	1.2463 × 10^2^
*η*	−0.0030	3.2885 × 10^2^
*δ*_1_	−6.7225 × 10^−4^	1.4875 × 10^3^
*δ*_2_	−3.1119 × 10^−4^	3.2134 × 10^3^
*δ*_3_	−1.5198 × 10^−4^	6.5799 × 10^3^

## Data Availability

The case data used to support the findings of this study are published on the Hubei Provincial Health Commission website, which we can obtain by visiting http://wjw.hubei.gov.cn/bmdt/dtyw/. These network direct reporting data are completely public, and we count these data day by day.

## References

[1] WHO *Naming the coronavirus disease (COVID-19) and the virus that causes it*.

[2] The State Council *The People’s Republic of China. Fighting COVID-19: China in action*.

[3] Health Commission of Hubei Province *Epidemic Situation of COVID-19 in Hubei Province on April 16, 2020*.

[4] Health Commission of Hubei Province *Epidemic Situation of COVID-19 in Hubei Province on April 26, 2020*.

[5] Chinanews *Wuhan eyes 10-day city-wide testing for all residents*.

[6] National Health Commission of the People’s Republic of China *Notice of the general office of the National Health Commission on issuing the New Coronavirus Pneumonia Prevention and Control Plan*.

[7] The State Council The People’s Republic of China *Notice of the general office of the National Health Commission on issuing the New Coronavirus Pneumonia Prevention and Control Plan (Trial Version 5)*.

[8] Dietz K. (1993). The estimation of the basic reproduction number for infectious diseases. *Statistical Methods in Medical Research*.

[9] Collins O. C., Duffy K. J. (2020). Estimating the impact of lock-down, quarantine and sensitization in a COVID-19 outbreak: lessons from the COVID-19 outbreak in China. *Peer J*.

[10] Yi H., Wang K., Wang W. (2020). Analysis of transmissibility of COVID-19 and regional differences in disease control (in Chinese). *Acta Mathematicae Applicatae Sinica*.

[11] Mizumoto K., Kagaya K., Chowell G. (2020). Early epidemiological assessment of the transmission potential and virulence of coronavirus disease 2019 (COVID-19) in Wuhan City, China, January–February, 2020. *BMC Medicine*.

[12] Jung S.-m., Akhmetzhanov A. R., Hayashi K. (2020). Real-time estimation of the risk of death from novel coronavirus (COVID-19) infection: inference using exported cases. *Journal of Clinical Medicine*.

[13] Riou J., Althaus C. L. (2020). Pattern of early human-to-human transmission of Wuhan 2019 novel coronavirus (2019-nCoV), December 2019 to January 2020. *Eurosurveillance*.

[14] Li Q., Guan X., Wu P. (2020). Early transmission dynamics in Wuhan, China, of novel coronavirus–infected pneumonia. *New England Journal of Medicine*.

[15] Zhao S., Lin Q., Ran J. (2020). Preliminary estimation of the basic reproduction number of novel coronavirus (2019-nCoV) in China, from 2019 to 2020: A data-driven analysis in the early phase of the outbreak. *International Journal of Infectious Diseases*.

[16] Sun G., Wang S., Li M., Li L., Feng G. (2020). Transmission dynamics of COVID-19 in Wuhan, China: effects of lockdown and medical resources. *Nonlinear Dynamics*.

[17] Pang L., Liu S., Zhang X., Tian T., Zhao Z. (2020). Transmission dynamics and control strategies of COVID-19 in Wuhan, China. *Journal of Biological Systems*.

[18] Chinazzi M., Davis J. T., Ajelli M., Gioannini C., Vespignani A. (2020). The effect of travel restrictions on the spread of the 2019 novel coronavirus (COVID-19) outbreak. *Science*.

[19] Tian H., Liu Y., Li Y. (2020). An investigation of transmission control measures during the first 50 days of the COVID-19 epidemic in China. *Science*.

[20] Wan H., Cui J., Yang G. (2020). Risk estimation and prediction of the transmission of coronavirus disease-2019 (COVID-19) in the mainland of China excluding Hubei province. *Infectious Diseases of Poverty*.

[21] Zhang B., Zhou H., Zhou F. (2020). Study on SARS-CoV-2 transmission and the effects of control measures in China. *PLoS One*.

[22] Li Y., Wang L., Peng Z., Shen H. (2020). Basic reproduction number and predicted trends of coronavirus disease 2019 epidemic in the mainland of China. *Infectious Diseases of Poverty*.

[23] Jia J., Ding J., Liu S. (2020). Modeing the control of COVID-19: impact of policy interventions and meteorological factors. *Electronic Journal of Differential Equations*.

[24] Tang B., Wang X., Li Q. (2020). Estimation of the transmission risk of the 2019-nCoV and its implication for public health interventions. *Journal of Clinical Medicine*.

[25] Song H., Li F., Jia Z., Jin Z., Liu S. (2020). Using traveller-derived cases in Henan province to quantify the spread of COVID-19 in Wuhan, China. *China Nonlinear Dynamics*.

[26] Wang L., Wang J., Zhao H. (2020). Modelling and assessing the effects of medical resources on transmission of novel coronavirus (COVID-19) in Wuhan, China. *China. Mathematical Biosciences and Engineering*.

[27] Abbott S., Hellewell J., Munday J., Funk S. (2020). The transmissibility of novel coronavirus in the early stages of the 2019-20 outbreak in Wuhan: exploring initial point-source exposure sizes and durations using scenario analysis. *Research*.

[28] Read J. M., Bridgen J. R. E., Cummings D. A. T., Ho A., Jewell C. P. (2021). Novel coronavirus 2019-nCoV (COVID-19): early estimation of epidemiological parameters and epidemic size estimates. *Philosophical Transactions of the Royal Society B*.

[29] Ndaïrou F., Area I., Nieto J. J., Torres D. F. M. (2020). Mathematical modeling of COVID-19 transmission dynamics with a case study of Wuhan. *Chaos, Solitons & Fractals*.

[30] Li R., Pei S., Chen B. (2020). Substantial undocumented infection facilitates the rapid dissemination of novel coronavirus (SARS-CoV-2). *Science*.

[31] Xue L., Jing S., Miller J. C. (2020). A data-driven network model for the emerging COVID-19 epidemics in Wuhan, Toronto and Italy. *Mathematical Biosciences*.

[32] Zhanwei D., Xu X., Wu Y., Wang L., Cowling B. J., Meyers L. A. (2020). Serial interval of COVID-19 among publicly reported confirmed cases. *Emerging Infectious Diseases*.

[33] WHO WHO Statement on the first meeting of the International Health Regulations. https://www.who.int/news-room/detail/23-01-2020-statement-on-the-meeting-of-the-international-health-regulations-%282005%29-emergency-committee-regarding-the-outbreak-of-novel-coronavirus-%282019-ncov%29.

[34] Zhao S., Musa S. S., Lin Q. (2020). Estimating the unreported number of novel coronavirus (2019-nCoV) cases in China in the first half of January 2020: a data-driven modelling analysis of the early outbreak. *Journal of Clinical Medicine*.

[35] Liu J., Zhou Y., Ye C., Zhang G., Zhang F., Song C. (2021). The spatial transmission of SARS-CoV-2 in China under the prevention and control measures at the early outbreak. *Archives of Public Health*.

[36] Wu J. T., Leung K., Leung G. M. (2020). Nowcasting and forecasting the potential domestic and international spread of the 2019-nCoV outbreak originating in Wuhan, China: a modelling study. *The Lancet*.

[37] Guo X.-J., Zhang H., Zeng Y.-P. (2020). Transmissibility of COVID-19 in 11 major cities in China and its association with temperature and humidity in Beijing, Shanghai, Guangzhou, and Chengdu. *Infectious Diseases of Poverty*.

[38] Musa Salihu S., Gao D., Zhao S., Yang L., Lou Y., He D. (2020). Mechanistic modeling of the coronavirus disease 2019 (COVID-19) outbreak in the early phase in Wuhan, China, with different quarantine measures (in Chinese). *Acta Mathematicae Applicatae Sinica*.

[39] Zhou T., Liu Q., Yang Z. (2020). Preliminary prediction of the basic reproduction number of the Wuhan novel coronavirus 2019-nCoV. *Journal of Evidence-Based Medicine*.

[40] Huang S., Jin Z., Peng Z. (2020). Studies of the strategies for controlling the COVID-19 epidemic in China: estimation of control efficacy and suggestions for policy makers (in Chinese). *Scientia Sinica Mathematica*.

[41] Ma Y., Liu X., Tao W. (2020). Estimation of the outbreak severity and evaluation of epidemic prevention ability of COVID-19 by province in China. *American Journal of Public Health*.

[42] Chen T., Rui J., Wang Q., Zhao Z., Cui J., Yin L. (2020). A mathematical model for simulating the phase-based transmissibility of a novel coronavirus. *Infectious Diseases of Poverty*.

[43] Wang X., Tang S., Chen Y., Feng X., Xiao Y., Zongben X. (2020). When will be the resumption of work in Wuhan and its surrounding areas during COVID-19 epidemic? A data-driven network modeling analysis. *Scientia Sinica Mathematica*.

[44] Song P., Lou Y., Zhu L., Li W., Jiang H. (2020). Multi-stage and multi-scale patch model and the case study of novel coronavirus (in Chinese). *Acta Mathematicae Applicatae Sinica*.

[45] Sanche S., Lin Y. T., Xu C., Romero-Severson E., Hengartner N., Ke R. (2020). High contagiousness and rapid spread of severe acute respiratory syndrome coronavirus 2. *Emerging Infectious Diseases*.

[46] Wang K., Zhenzhen L., Wang X. (2020). Current trends and future prediction of novel coronavirus disease (COVID-19) epidemic in China: a dynamical modeling analysis. *Mathematical Biosciences and Engineering*.

[47] Anderson R. M., Heesterbeek H., Klinkenberg D., Déirdre T., Hollingsworth (2020). How will country-based mitigation measures influence the course of the COVID-19 epidemic?. *The Lancet*.

[48] Artiono R., Prawoto B. P., Hidayat D., Yunianti D. N., Astuti Y. P. (2020). The dynamics of COVID-19: the effect of largescale social restrictions. *Communications in Mathematical Biology and Neuroscience*.

[49] Health Commission of Hubei Province *Epidemic Situation of COVID-19 in Hubei Province on April 17, 2020*.

[50] USA Today How coronavirus spreads so quickly and how you can slow it down. How coronavirus spreads so quickly and how you can slow it down. https://www.usatoday.com/pages/interactives/news/coronavirus-covid-spread-quickly-how-to-slow-it-down/.

[51] Diekmann O., Heesterbeek J. A. P., Metz J. A. J. (1990). On the definition and the computation of the basic reproduction ratio *ℛ*_0_ in models for infectious diseases in heterogeneous populations. *Journal of Mathematical Biology*.

[52] van den Driessche P., Watmough J. (2002). Reproduction numbers and sub-threshold endemic equilibria for compartmental models of disease transmission. *Mathematical Biosciences*.

[53] Health Commission of Hubei Province *Situation reports*.

[54] WHO *Coronavirus disease (COVID-2019) situation reports*.

[55] Lauer S. A., Grantz K. H., Bi Q. (2020). The incubation period of coronavirus disease 2019 (COVID-19) from publicly reported confirmed cases: estimation and application. *Annals of Internal Medicine*.

[56] Chowell G., Fenimore P. W., Castillo-Garsow M. A., Castillo-Chavez C. (2003). SARS outbreaks in Ontario, Hong Kong and Singapore: the role of diagnosis and isolation as a control mechanism. *Journal of Theoretical Biology*.

[57] People’s Government of Hubei Province *Statistical Communique on Hubei province’s national economic and social development in 2019*.

[58] National Health Commission of the People’s Republic of China *Interpretation of the Pneumonia Diagnosis and Treatment Plan for Novel Coronavirus Infection (Trial Version 5)*.

[59] Zhang X., Zhao Y., Neumann A. U. (2010). Partial immunity and vaccination for influenza. *Journal of Computational Biology*.

[60] Li Y., Wang L., Pang L., Liu S. (2016). The data fitting and optimal control of a hand, foot and mouth disease (HFMD) model with stage structure. *Applied Mathematics and Computation*.

[61] Samsuzzoha M., Singh M., Lucy D. (2013). Uncertainty and sensitivity analysis of the basic reproduction number of a vaccinated epidemic model of influenza. *Applied Mathematical Modelling*.

[62] Lipsitch M., Cohen T., Cooper B. (2003). Transmission dynamics and control of severe acute respiratory syndrome. *Science*.

[63] Beingjing Municipal Health Commission *Beijing reported 36 COVID-19 cases yesterday*.

[64] Health Commission of Xinjiang province *Epidemic situation of novel coronavirus pneumonia in Xinjiang on 28 October 2020*.

[65] Health Commission of Liaoning province *Epidemic situation of novel coronavirus pneumonia in Liaoning on 29 December 2020*.

[66] Health Commission of Hebei Province *Epidemic situation of novel coronavirus pneumonia in Hebei on 12 January 2021*.

[67] Health Commission of Heilongjiang province Latest epidemic situation report. http://wsjkw.hlj.gov.cn/pages/5fff819912c15b4f6dce6a68.

[68] Health Commission of Jilin Province *Bulletin of COVID-19 epidemic situation of Health Commission of Jilin Province (published on 20 January 2021)*.

[69] Jiangsu Commission of Health *Epidemic situation of novel coronavirus pneumonia in Jiangsu on 5 August 2021*.

[70] Health Commission of Henan Province *Epidemic situation of novel coronavirus pneumonia in Henan on 8 August 2021*.

[71] Fujian Provincal Health Commission *Epidemic Situation of Novel Coronavirus Pneumonia in Fujian on 16*.

[72] National Health Commission of the People’s Republic of China *Epidemic situation of novel coronavirus pneumonia in Inner Mongolia on 26 October 2021*.

[73] National Health Commission of the People’s Republic of China *How to make personal protection?*.

[74] National Health Commission of the People’s Republic of China *COVID-19 Protection Guidebook*.

[75] Gao Z., Yinghui X., Chao Sun X., Wang Y. G., Qiu S., Ma K. (2021). A systematic review of asymptomatic infections with COVID-19. *Journal of Microbiology, Immunology and Infection*.

[76] WHO *COVID-19 vaccines*.

[77] Russell T. W., Wu J. T., Sam Clifford W., Edmunds J., Kucharski A. J., Jit M. (2021). Effect of internationally imported cases on internal spread of COVID-19: a mathematical modelling study. *The Lancet Public Health*.

[78] Zhao Y., Huang J., Zhang L., Chen S., Gao J., Jiao H. (2022). The global transmission of new coronavirus variants. *Environmental Research*.

[79] Serhani M., Labbardi H. (2021). Mathematical modeling of COVID-19 spreading with asymptomatic infected and interacting peoples. *Journal of Applied Mathematics and Computing*.

[80] Boldog P., Tekeli T., Vizi Z., Dénes A., Bartha F. A., Röst G. (2020). Risk assessment of novel coronavirus COVID-19 outbreaks outside China. *Clinical Medicine*.

